# Protocol for assessing BMP9-induced angiogenic and osteogenic activity *in vitro* and *in vivo*

**DOI:** 10.1016/j.xpro.2026.104894

**Published:** 2026-10-13

**Authors:** Jianing Duan, Yuanyuan Zhang, Weida Zhang, Xiaohong Qin, Li-Zhi Mi

**Affiliations:** 1 School of Life Sciences, Faculty of Medicine, Tianjin University, Tianjin 300072, P.R. China; 2 State Key Laboratory of Synthetic Biology, Tianjin University, Tianjin 300072, P.R. China

**Keywords:** Cell Biology, Developmental biology, Protein Biochemistry

## Abstract

BMP9 is an important regulator in angiogenesis, tissue development, and immune response. Here, we present a protocol to examine the regulatory mechanism of unprocessed BMP9 precursor in BMP9 signaling. We describe procedures for assessing BMP9-induced angiogenic and osteogenic activity *in vitro* and *in vivo*. We describe steps for their assessment using flow cytometric analysis, C2C12 differentiation assay, chicken embryo chorioallantoic membrane assay, and mouse subcutaneous Matrigel assay.

For complete details on the use and execution of this protocol, please refer to Zhang et al.^1^

## Highlights

• Analyze competitive inhibition of BMP9 precursor to its receptors by flow cytometry
• Measure inhibitory activity of BMP9 precursor on C2C12 cell osteogenic differentiation
• Analyze angiogenic activities of BMP9 and its precursor with chicken embryo CAM assay
• Analyze the angiogenic activities of BMP9s using an *in vivo* Matrigel plug assay

## Abstract (editor highlights)

Publisher’s note: Undertaking any experimental protocol requires adherence to local institutional guidelines for laboratory safety and ethics.

## Graphical abstract

**Figure:**
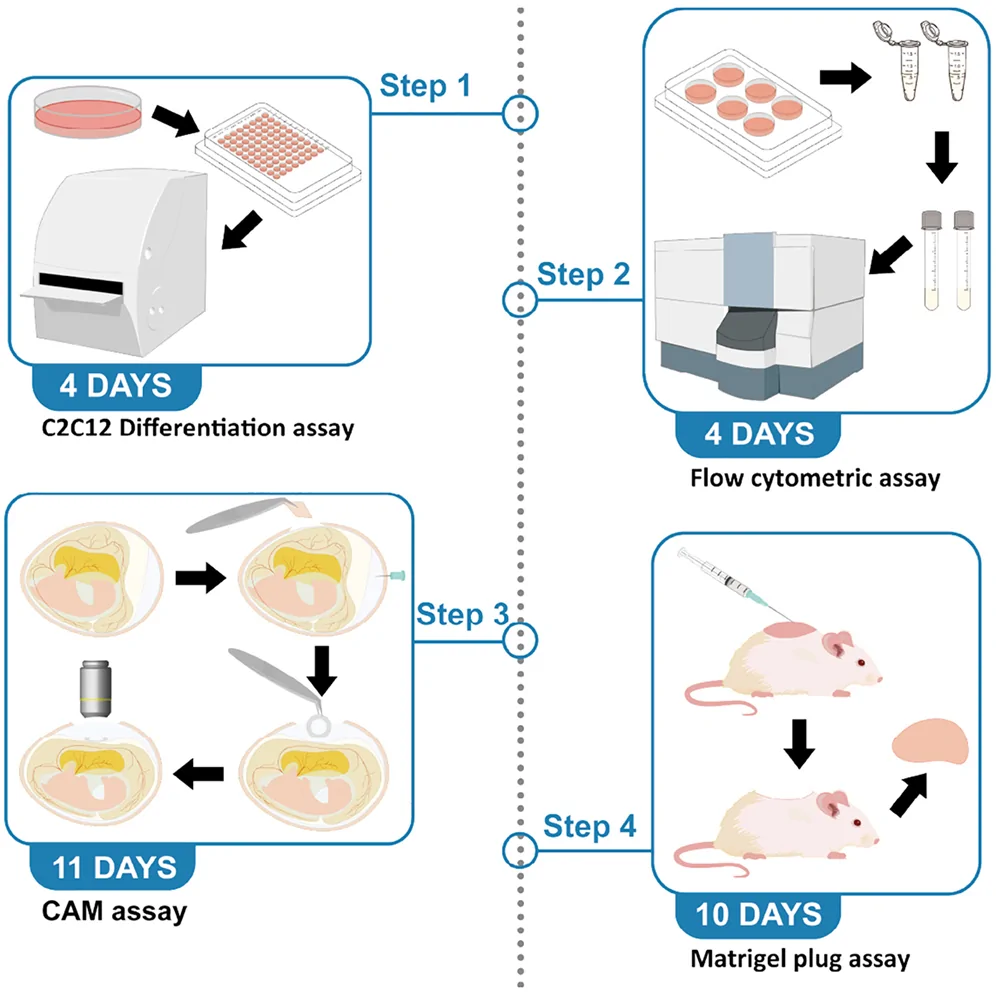

## Before you begin

This protocol describes the specific steps to analyze: (1) the competition between Pro-BMP9 and BMP9 precursor for binding to their cognate receptors by flow cytometry, (2) the inhibitory mechanism of BMP9 precursor on Pro-BMP9-induced C2C12 differentiation, (3) the angiogenic activities of Pro-BMP9 and BMP9 precursor with the chicken embryo chorioallantoic membrane assay, and (4) the regulation of BMP9 signaling by BMP9 precursor using the Matrigel plug assay.

### Innovation

This protocol establishes a standardized workflow for evaluating the inhibitory effects of BMP9 precursor on BMP9 signaling in osteogenesis and angiogenesis. The workflow integrates flow cytometry, C2C12 alkaline phosphatase (ALP) activity assay, chicken embryo chorioallantoic membrane (CAM) assay, and Matrigel plug assay, enabling reproducible assessment under standardized conditions. This workflow provides a practical platform for studying the regulatory mechanism of unprocessed BMP9 precursor in BMP9 signaling and can be adapted for functional characterization of other TGF-β family members.

### Institutional permissions

All animal experiments were conducted in accordance with the regulations of the Institute of Radiation Medicine of the Chinese Academy of Medical Sciences; and the experiment was approved by the Institutional Ethics Committee (IRM-DWLL-2018069).

### Cell culture

**Timing: 5–7 days**

The protocol below describes the specific steps for culturing HEK293T and C2C12 cells, which will be used for the following experiments.

1. Cell thawing
  a. Pre-warm the water bath for 10 min before incubating Dulbecco’s Modified Eagle Medium (DMEM) supplemented with 10% Fetal Bovine Serum (FBS) in the water bath.
  b. Disinfect the outer surface of bottles and culture dishes with 75% ethanol.
  c. Place the bottles and culture dishes in biosafety cabinet.
  d. Put the cryogenic vials into the water bath, and shake the vials to accelerate thawing.
  e. Disinfect the cryogenic vials with 75% ethanol.
  f. Transfer thawed cells into a 15 mL centrifuge tube prefilled with 3–5 mL pre-warmed DMEM medium supplemented with 10% serum in a biosafety cabinet.
  g. Centrifuge the tube at 144 × *g* for 5 min, discard the supernatant, and add 3 mL culture medium to resuspend cells.
  h. Aliquot the cells to 3.5 cm dishes.
  i. Incubate cells at 37°C for 1–2 days in a 5% CO_2_ incubator until the cells reach 70–80% confluency.

***Note:*** The cryopreservation medium contains DMSO which is toxic to cells.

**CRITICAL:** It’s important to ensure that the vial lid is not immersed in water during shaking in water bath to avoid contamination.

2. Cell passage.
  a. Discard the culture medium.
  b. Gently rinse the cells with 1 mL of PBS pre-warmed to 37°C in a water bath.
  c. Discard PBS and then add 0.5 mL of 0.25% Trypsin solution (with EDTA).
  d. Incubate the cells in Trypsin solution for 1–2 min to digest and detach the cells.
  e. Add 3–5 mL complete medium to stop the digestion and gently pipette medium to disperse the cells.
  f. Transfer cell suspension to 10 cm dishes (1 mL per dish), then add complete medium at subculture ratio of 1:3–1:5.
  g. Ensure the cells are evenly distributed and then incubate the cells at 37°C, 5% CO_2_.

***Note:*** Duration for incubation the cells in Trypsin solution depends on the type of cells.

**CRITICAL:** C2C12 cells should be subcultured at a passage ratio of 1:8–1:10 before reaching 70% confluency to prevent their differentiation into osteogenic cells.

3. Cryopreservation.
  a. Cryopreserve cells upon 70–80% confluency.
  b. Discard the culture medium, and wash cells once with 1 mL of PBS.
  c. Discard PBS and digest cells with 0.5 mL of 0.25% Trypsin solution for 1–2 min.
  d. Stop the digestion by adding 3–5 mL of DMEM supplemented with 10% serum.
  e. Aspirate cells by gently pipetting.
  f. Transfer cell suspension to a 15 mL centrifuge tube, and centrifuge the tube at 144 × *g* for 5 min.
  g. Discard medium and resuspend cells in 5 mL of cryopreservation medium.
  h. Transfer cell suspension to cryogenic vials (1 mL per vial) and place them in a pre-cooled freezing container at −80°C before transferring them to liquid nitrogen.

***Note:*** Perform controlled-rate freezing to prevent the formation of intracellular ice.

### Cell preparation

**Timing: 1–2 days**

4. Seeding C2C12 cells for differentiation assay.
  a. Discard the culture medium and detach the cells by following the procedure described in step 2.
  b. Collect the cells and then seed them at a density of 2 × 10^4^ cells per well into 96-well plates, bring the final volume to 200 μL per well, ensuring the cells are evenly distributed.
  c. Incubate the plates at 37°C, 5% CO_2_ for approximately 14 h.
5. Plasmid transfection into HEK293T cells for flow cytometric analysis.
  a. Detach the cells following the procedure described in step 2.
  b. Collect and seed 2 × 10^5^ cells per well into 6-well plates, and add complete medium to bring the total volume to 2 mL per well.
  c. Ensure the cells are evenly distributed and then incubate them at 37°C, 5% CO_2_ until the cells reach 70–80% confluency (approximately 18–24 h).
  d. Transfect cells with plasmids using Polyethylenimine (PEI). For HEK293T cells, dilute 5 μg of the plasmids encoding HA-tagged ALK1 and/or Flag-tagged ENG in PBS to a final volume of 50 μL each, dilute 15 μL of PEI stock solution (1 mg/mL) to a final volume of 50 μL using PBS, then add the PEI solution dropwise to the plasmid solution.
  e. Incubate the mixture for 10 min at 22°C–25°C and then add the plasmids to the cells in droplets.
  f. After 6 h of post-transfection, replace the culture medium with fresh complete medium.

***Note:*** Add the PEI solution dropwise to the DNA solution and mix the solution immediately to ensure the homogeneous formation of nanocomplex.

**CRITICAL:** Heat plasmids at 70°C for 0.5–1 h to reduce the risk of contamination after they are extracted.

### Chicken embryo preparation

**Timing: 8 days**

6. Incubation of specific pathogen free (SPF) eggs.
  a. Clean the incubator and wipe thoroughly with 75% ethanol.
  b. Clean the received SPF eggs with sterile water to remove surface debris.
  c. Allow the eggs to air-dry completely, spray the eggs with 75% ethanol, and then allow the ethanol to evaporate before incubation.
  d. Place the egg trays into the incubator with the eggs positioned horizontally. Incubate them at 37.8°C with 60%–65% humidity for 5 days.
  e. Gently rotate the eggs 3 times each day.
  f. Check the eggs with an egg candler and discard unfertilized eggs.
  g. Incubate the remaining eggs for an additional 3 days. (Figure 4B) See troubleshooting – problem 1.

***Note:*** Rotate the eggs routinely to imitate natural incubation.

## Key resources table

**Table:** 

REAGENT or RESOURCE	SOURCE	IDENTIFIER
**Antibodies**		

Anti-HA-tag Antibody (Mouse Monoclonal) (1:3000)	TransGen Biotech	Cat #: HT301-01; RRID: AB_3076171
Anti-Flag-tag Antibody (1:3000)	Zhang et al.^1^	N/A; RRID: AB_3696579
ALFA-nanobody-EGFP (0.23 μg/mL)	Zhang et al.^1^	N/A; RRID: AB_3696587
Anti-CD31 Antibody (1:2000)	Fuzhou Maixin Biotech	MAB-0031
Goat anti-Mouse IgG(H+L)-FITC (1:200)	Bioss	bs-0296G-FITC

**Chemicals, peptides, and recombinant proteins**		

Polyethylenimine (PEI)	Polysciences	Cat #: 23966
Dulbecco’s Modified Eagle Medium (DMEM)	VivaCell	Cat #: C3113-0500
Trypsin	Gibco	Cat #: 25200056
Fetal Bovine Serum (FBS)	Biological Industries	Cat #: 04-001-1ACS
Serum-Free Cell Freezing Medium	Yoche Biotech	Cat #: YC0100
BSA	biosharp	Cat #: BS114
P-Nitrophenyl phosphate (pNPP)	Thermo Scientific	Cat #:34045
Glycine	Sangon Biotech	Cat #: A502065-0005
Triton X-100	Solarbio	Cat #: T8200
MgCl_2_-6H_2_O	Sigma	Cat #: 63064
NaOH	Sigma	Cat #: 484024
PBS	VivaCell	Cat #: C3593-0500
Matrigel	Corning	Cat #: 356234
Pro-BMP9 (1–429)	Zhang et al.^1^	N/A
Precursor (1–429)	Zhang et al.^1^	N/A
Acetone	Sigma	Cat #: 179124
Methanol	Sigma	Cat #: 34860-1L-R
Formaldehyde solution	Sigma	Cat #: F8775
Sodium heparin	MedChemExpress	Cat #: HY-17567A
Xylene	Sigma	Cat #: 534056
Hematoxylin-Eosin (HE) Stain Kit	Solarbio	Cat #: G1120
Neutral balsam	Solarbio	Cat #: G8590
Envision-plus HRP-labelled Dextran polymer	Invitrogen	Cat #: polymer 87–8973
DAB staining solution	Fuzhou Maixin Biotech	Cat #: DAB-2031
Counterstain hematoxylin	Sigma	Cat #: 51275

**Experimental models: Cell lines**		

HEK293T; *Homo sapiens*; genotype: N/A; age: N/A; sex: N/A	National Collection of Authenticated Cell Cultures	Cat #: 3101HUMSCSP502
C2C12; *Mus musculus*; genotype: wild type; age: N/A; sex: N/A	National Collection of Authenticated Cell Cultures	Cat #: 4201MOU-CCTCC00175

**Experimental models: Organisms/strains**		

chicken embryo; *Gallus gallus domesticus*; age: N/A; sex: N/A	Zhang et al.^1^	SPF White Leghorn
Mouse; *Mus musculus*; strain: BALB/c; age: 6–8 weeks; sex: 20 females and 20 males	Zhang et al.^1^	BALB/c

**Recombinant DNA**		

ET3-HA-ALK1(1–482) (plasmid)	Zhang et al.^1^	N/A
pEF1-Flag-ENG (1–633) (plasmid)	Zhang et al.^1^	N/A

**Software and algorithms**		

Origin	OriginLab Corporation	https://www.originlab.com/
ImageJ	Schneider et al.,^2^	https://imagej.nih.gov/ij/
FlowJo	Thomas A Ujas.,^3^	https://www.flowjo.com/

**Other**		

CO_2_ Incubator	Thermo Fisher Scientific	HERAcell 150i
Olympus IX73 microscope	Olympus	IX73
EnVision Multilabel Reader	PerkinElmer	EnVision
BD FACSCalibur	Becton Dickinson	FACSCalibur
High-speed Centrifuge 5810R	Thermo Fisher Scientific	5810R
Egg Incubator	Dezhou Weiqian Import And Export Co., Ltd	G-2048
Silicone rings (6∗0.3 mm)	Gelisen	N/A

## Materials and equipment

**Table:** PEI solution

Reagent	Final concentration	Amount or volume
Polyethylenimine (PEI)	1 mg/mL	0.1 g
ddH_2_O	N/A	To 100 mL
**Total**	**N/A**	**100 mL**

***Note:*** PEI solution should be adjusted to pH 7.2–7.4 and stored at −20°C for up to 1 year.

**Table:** Alkaline phosphatase test solution

Reagent	Final concentration	Amount or volume
pNPP	19 mM	105 mg
Glycine	0.1 M (pH 10.3)	187.6 mg
MgCl_2_-6H_2_O	17 mM	85 mg
Triton X-100	0.1%	25 μL
ddH_2_O	N/A	To 25 mL
**Total**	**N/A**	**25 mL**

***Note:*** Alkaline phosphatase test solution needs to be prepared in fresh.

**Table:** CAM fixation solution

Reagent	Final concentration	Amount or volume
Acetone	50% (v/v)	50 mL
methanol	50% (v/v)	50 mL
**Total**	**N/A**	**100 mL**

***Note:*** CAM fixation solution needs to be prepared fresh.

## Step-by-step method details

### Flow cytometric assay

**Timing: 1 day**

The following steps describe the detailed procedures for flow cytometric analysis.

1. For the analysis of BMP9 precursor binding to its receptors, dilute ALFA-tagged BMP9 precursor to a final concentration of 0.2 μM in serum-free DMEM.
2. For the analysis of BMP9 precursor and Pro-BMP9 competition, prepare mixtures containing a final concentration of 0.4 μM ALFA-tagged BMP9 precursor and 0–2 μM Pro-BMP9 in serum-free DMEM.
3. Harvest the cells.
  a. Discard the culture medium, rinse the cells with PBS and detach the cells with Trypsin.
  b. Add 1 mL DMEM supplemented with 10% FBS to stop the digestion.
  c. Resuspend the cells and transfer them to microcentrifuge tubes.
  d. Centrifuge at 420 × *g* for 5 min.
4. Prepare the cells for flow cytometric analysis.
  a. Discard the supernatant, resuspend the cells with 1 mL PBS.
  b. Centrifuge them at 420 × *g* for 3 min. Repeat this step 3 times.
  c. Resuspend the cells in 200 μL PBS and split them into two halves.
  d. Incubate 100 μL cells with anti-HA antibody (1:3000) or anti-Flag antibody (1:3000) according to the manufacturer’s instructions on a shaker at 4°C for 1 h.
  e. Incubate the rest 100 μL cells with 0.2 μM ALFA-tagged BMP9 precursor (binding assay) or a protein mixture containing 0.4 μM ALFA-tagged BMP9 precursor and 0–2 μM Pro-BMP9 (competition assay) on a shaker at 4°C for 1 h.
  f. Wash the cells once with PBS and then incubate them separately with FITC-labelled secondary antibody (1:200, for analysis of receptor expression) or ALFA-nanobody-EGFP (0.23 μg/mL, for analysis of BMP9 precursor binding) on a shaker at 4°C for 1 h.
  g. Discard the staining solution and wash the cells with PBS as sub-step a.
  h. Resuspend the cells in microcentrifuge tubes with 500 μL of PBS.
  i. Filter the cell suspension with a 200-mesh nylon strainer to remove cell clumps.
  j. Keep the tubes on ice for later use (Figure 1).

**Figure 1:**
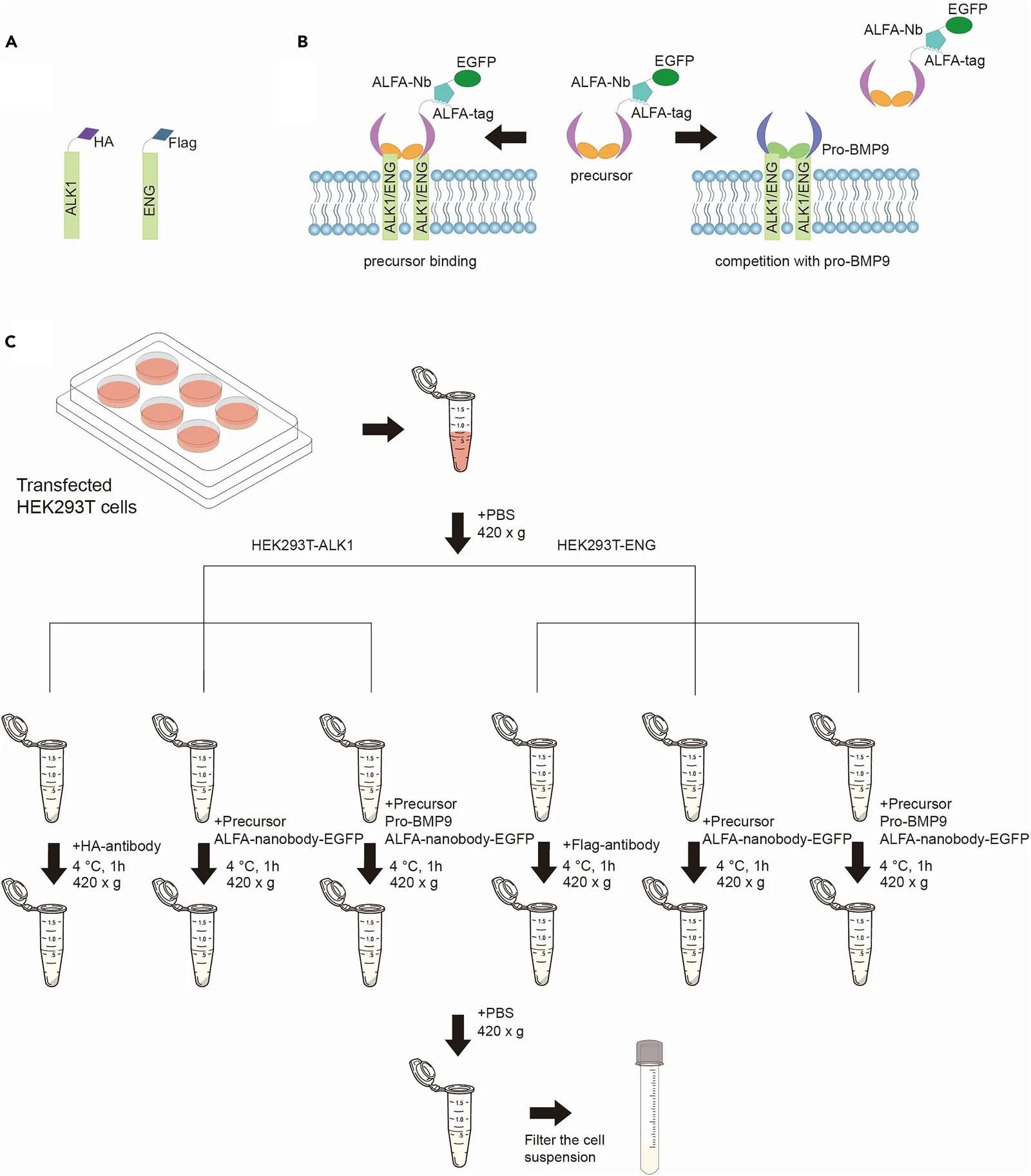
Schematic diagram of the flow cytometry experimental process (A) Schematic of the HA-tagged ALK1 and Flag-tagged ENG constructs. (B) Schematic diagram of precursor binding to ALK1 and/or ENG expressed on HEK293T cells and its competition with pro-BMP9. (C) Workflow of the flow cytometric assay. Cells were harvested and washed with PBS, and then incubated separately with anti-HA antibody (1:3000) (receptor expression), anti-Flag antibody (1:3000) (receptor expression), 0.2 μM ALFA-tagged BMP9 precursor (binding assay), or a protein mixture containing 0.4 μM ALFA-tagged BMP9 precursor and 0–2 μM Pro-BMP9 (competition assay). After washing, the cells were incubated with FITC-labelled secondary antibody (1:200, for analysis of receptor expression) or ALFA-nanobody-EGFP (0.23 μg/mL, for analysis of the precursor binding) before flow cytometric analysis.

***Note:*** One part of the cells is used for analyzing receptor expression, while the other is used for analyzing the binding of BMP9 precursor.

***Note:*** Throughout this protocol, all listed protein concentrations refer to the final concentrations.

5. Perform the flow cytometric analysis on Calibur cytometer (BD).
  a. Turn on the cytometer and computer, launch the software, and communicate with the instrument.
  b. Ensure that the sheath tank is at least 75% full.
  c. Connect the computer to cytometer and set up a new document.
    i. Set x parameter to FSC-H 1024, and y parameter to SSC-H 1024.
    ii. Set Detector/Amps with FSC/SSC in linear mode and FL1 (FITC channel) in logarithmic mode.
    iii. Compensate for the fluorescent intensity as needed.
    iv. Save the setting file.
  d. Load negative control sample and adjust the FSC Amp Gain and the SSC Voltage to resolve the cell cluster clearly.
  e. Load test samples and verify the data file has been successfully stored before shutting down.
  f. Export the data and analyze them with FlowJo. See troubleshooting – problem 2.
6. Data analysis with FlowJo v10.8.
  a. Start FlowJo v10.8 and drag FCS files onto “All samples” to load the data files.
  b. Open the mock files (MOCK-FLAG/MOCK-HA) and set the x-axis to FSC-H and the y-axis to SSC-H.
  c. Identify the live cell population and exclude cell debris based on their FSC/SSC scattering distribution.
  d. Draw a suitable gate around the live cell population.
  e. Double-click the gate to open a new graph displaying the subpopulation.
  f. Use the histogram of the fluorescence from channel 1 (FITC channel) to distinguish the transfected or ligand-bound positive cells from the negative cells.
  g. Use the mock transfected samples to set the boundary of negative control in the gating.
  h. Ensure that the majority of negative events falls to the left of the threshold and then apply these settings to analyze the remaining samples (Figure 2).

**Figure 2:**
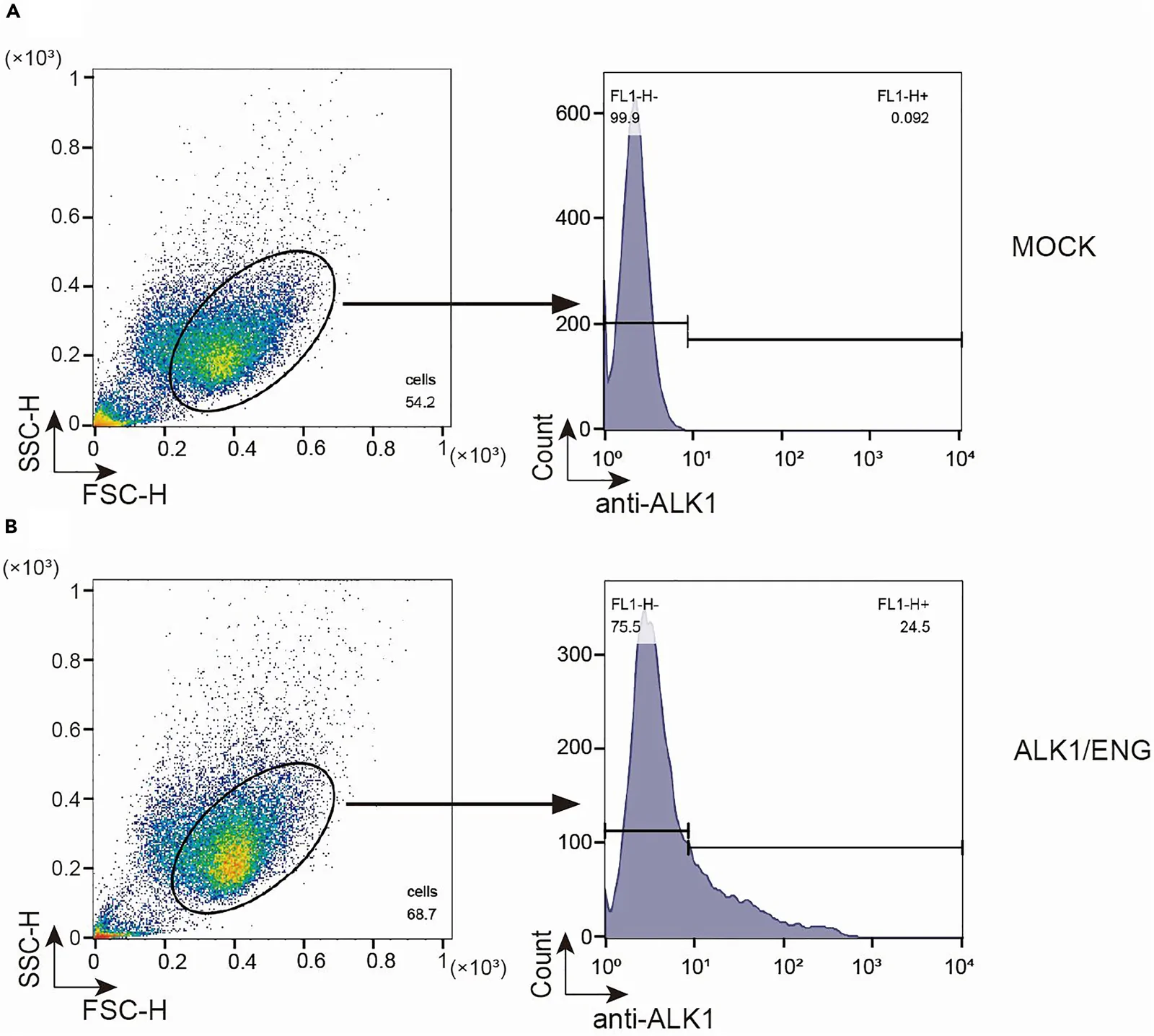
Gating strategy for flow cytometry analysis of transfected cell populations and precursor-bound cell populations (A) Negative control group (MOCK): Live cells were first identified using FSC-H and SSC-H values to exclude debris. A threshold was then set up using an interval gate on the FL1-H histogram. The boundary of negative population was set with the mock transfected sample. This gating strategy was consistently applied to all the samples. (B) Experimental group (ALK1/ENG): Following the same gating strategy as in (A).
  i. Quantify the percentage of positive cells and determine the mean and standard deviation for each group from triplicate biological repeats.
  j. Ensure that gating strategy remains identical across all samples and experiments to guarantee analytical consistency.

### C2C12 differentiation assay

**Timing: ∼4 days**

The following steps describe the detailed procedures for quantifying Pro-BMP9 and BMP9 precursor activities in osteogenesis using the C2C12 cells.

7. Determine the osteogenic activities of Pro-BMP9 and BMP9 precursor.
  a. Seed 2 × 10^4^ cells/well into 96-well plates, perform the C2C12 differentiation assay when the cells reach 70–80% confluency. It usually takes 14 h after seeding the cells.
  b. Perform a 12-point, two-fold serial dilution of Pro-BMP9 with DMEM supplemented with 1 mg/mL BSA (The final concentrations of Pro-BMP9 range from 80 nM to 0.039 nM) (Figure 3).

**Figure 3:**
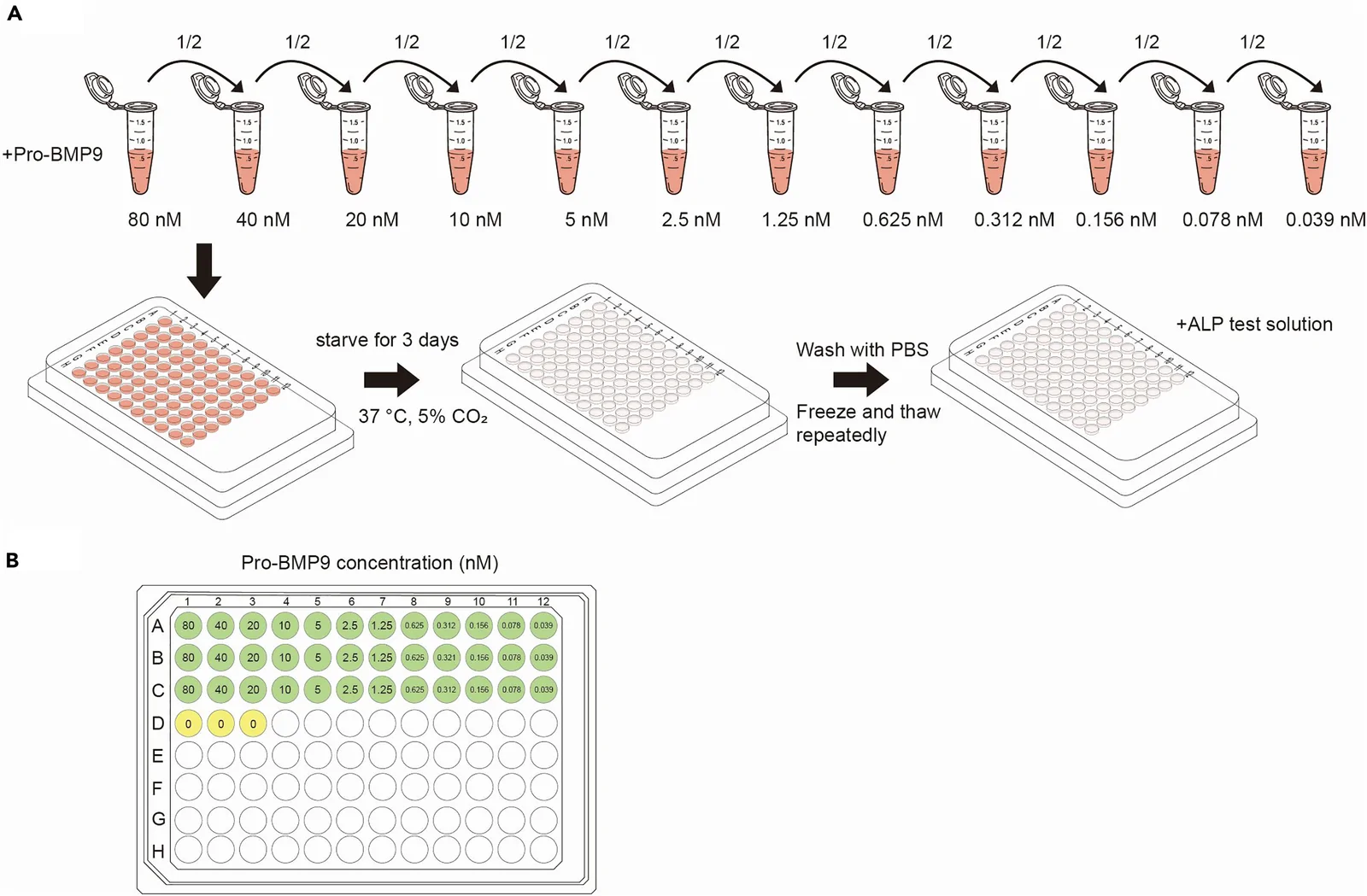
Workflow of the C2C12 differentiation assay and representation of the 96-well plate layout (A) A 12-point, two-fold serial dilution of Pro-BMP9 was performed using DMEM supplemented with 1 mg/mL BSA. Then the diluted solutions were added to the C2C12 cells in 96-well plates. After incubation for 3 days at 37°C in 5% CO_2_, the cells were washed with PBS and then frozen and thawed repeatedly to release Alkaline Phosphatase. After the ALP test solution was added to each well, the ALP activity of the cells was measured by using the EnVision Multilabel Reader. (B) Samples are loaded in triplicate from A1 to C12 (indicated in green). Blank controls are loaded in wells D1 to D3 as indicated in yellow.
  c. Discard the culture medium from 96-well plates.
  d. Gently rinse cells twice with pre-warmed DMEM (without serum).
  e. Add 100 μL per well of Pro-BMP9 or BMP9 precursor at different concentrations.
  f. Culture the cells at 37°C with 5% CO_2_ for 3 days.
  g. Wash the cells once with 100 μL of PBS per well, discard the PBS, then add 50 μL of ddH_2_O to each well.
  h. Freeze and thaw the cells in 96-well plates at −20°C and 22°C–25°C respectively for 3 times to release Alkaline Phosphatase.
  i. Add 50 μL of ALP test solution to each well.
  j. Shake the plate at 37°C for 30 min.
  k. Add 100 μL of 0.2 M NaOH to quench the reaction.
  l. Quantify the ALP activity of the cells by measuring the optical absorbance of the cells in each well at 405 nm using EnVision Multilabel Reader.
8. Quantify the inhibitory effect of BMP9 precursor on Pro-BMP9-induced osteogenic differentiation of C2C12 cells.
  a. Seed and culture the C2C12 cells as described in step 7-a.
  b. Perform a 12-point, two-fold serial dilution of BMP9 precursor with DMEM supplemented with 1 mg/mL BSA. (The final concentrations of BMP9 precursor range from 1.7 μM to 0.8 nM).
  c. Serially dilute BMP9 precursor solution to a final volume of 50 μL with addition of 10 nM Pro-BMP9 in each well.
  d. Culture the cells at 37°C with 5% CO_2_ for 3 days.
  e. Wash the cells once with 100 μL of PBS per well; then add 50 μL of ddH_2_O in each well.
  f. Freeze and thaw the cells in 96-well plates at −20°C and 22°C–25°C, respectively, for 3 times to release Alkaline Phosphatase.
  g. Add 50 μL of ALP test solution to each well; and shake the plates at 37°C for 30 min. Then add 100 μL of 0.2 M NaOH to quench the reaction.
  h. Quantify the ALP activity of the cells by measuring the optical absorbance at 405 nm using EnVision Multilabel Reader.

***Note:*** Triplicate biological repeats are performed for each experiment.

9. Use the EnVision Multilabel Reader to quantify Pro-BMP9 and BMP9 precursor activities.
  a. Turn on the instrument and the controlling computer, wait for the instrument to complete initialization and self-checking.
  b. Click “Load” to load the plate on the plate carrier. Set up a new protocol for measuring optical absorbance at 405 nm.
  c. Select plate type, measurement mode and repeats.
  d. Measure the absorbance of each well and export the data for analysis in Origin. See troubleshooting – problem 3.
  e. Start Origin, import the raw data, and fit the data with the Michaelis-Menten equation.
  f. Execute the fitting process until reaching convergence, typically within 6 iterations, and evaluate model reliability using the Adjusted R-square.
  g. Apply analysis of ANOVA to confirm model significance and report fitting parameters as the means ± SDs.

### Chicken embryo chorioallantoic membrane assay

**Timing: ∼4 days**

The following steps describe the detailed procedures for analyzing BMP9 precursor inhibition on pro-BMP9-mediated vascular quiescence.

10. Aspirate the air to create an artificial air sac within the egg (Figure 4).
  a. Select 8-day-old chicken embryos, identify and mark the blunt end region that has distinct cross-vessels.
  b. Disinfect the eggshells with paper towels sprayed with 75% ethanol.
  c. Poke the blunt side using a push pin to open a hole to the air sac, ensuring the membrane remains intact.
  d. Score the marked region using the grinding wheel.
  e. Gently remove a small piece of the eggshell without damaging the shell membrane.
  f. Pierce a tiny slit in the shell membrane with a syringe tip, avoiding damage the CAM or vessels.
  g. Apply some drops of sterile saline (≈100 μL) to the membrane, then gently displace the air sac using the dropper bulb. See troubleshooting – problem 4.

**Figure 4:**
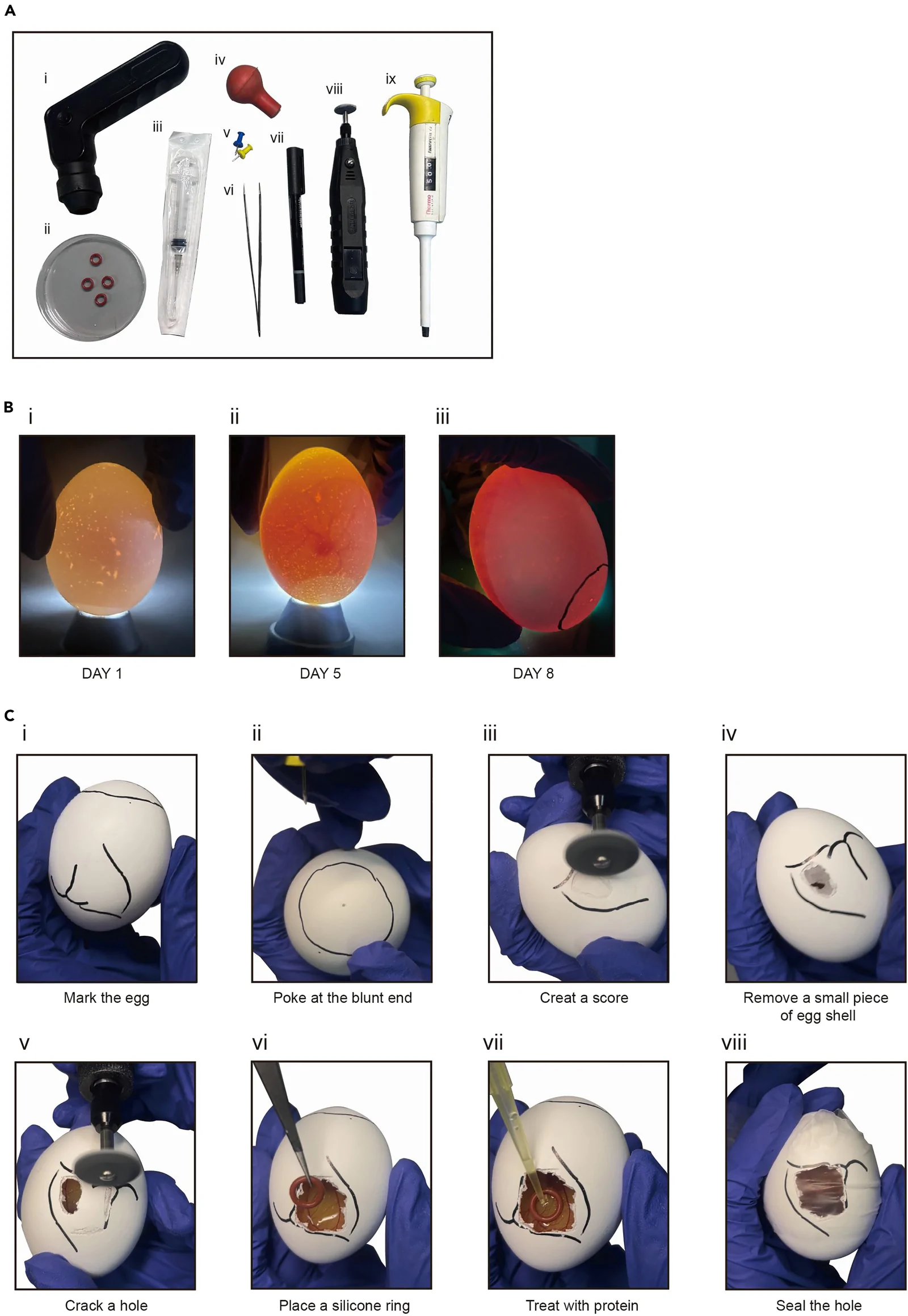
Procedures for CAM assay (A) Core equipment (i) egg candler, (ii) silicone rings, (iii) sterile tips, (iv) dropper bulb, (v) push pins, (vi) forceps, (vii) marker pen, (viii) micro-drill equipped with grinding wheel and (ix) micropipette. (B) The fertilized eggs at day 1(i), day 5(ii) and day 8(iii), showing the vessel’s formation during incubation. (C) CAM assay procedures. i) Mark the air sac and the blood vessels. ii) Poke the shell at the blunt end using a push pin. iii) Cut a score at the marked region using the grinding wheel. iv) Remove a small piece of the eggshell and pierce a tiny slit in the shell membrane, then displace the air sac using the dropper bulb. v) Crack a hole in the egg at the air chamber. vi) Place a silicone ring on the CAM. vii) Treat the CAM inside the silicone ring with proteins. viii) Seal the hole with Parafilm.
11. Apply FGF-2/Pro-BMP9/BMP9 precursor to the embryo.
  a. Crack a hole in each egg at the air chamber, place a silicone ring (6∗0.3 mm) on the CAM.
  b. Add 25 μL of 15 μM BSA, 22 nM FGF-2, 17.2 nM Pro-BMP9, 1.7 μM BMP9 precursor, or 17.2 nM Pro-BMP9 plus 1.7 μM BMP9 precursor inside the silicone ring. Each treatment was applied individually to 10 different eggs.
  c. Seal the hole with Parafilm or scotch tape, and incubate the eggs for an additional 3 days at 37.8°C.
12. Fix the CAM and analyze the angiogenesis using ImageJ.
  a. Remove the sealing parafilm.
  b. Apply the fix solution (acetone: methanol 1:1, v/v) to the CAM.
  c. Place the egg at 22°C–25°C for 20 min.
  d. Cut off the CAM inside the silicone ring.
  e. Place it on a culture dish. Visualize the CAM under a microscope.
  f. Analyze the number and morphology of blood vessels within the area enclosed by the silicone ring using ImageJ.
  g. Manually quantify newly formed vessels by excluding pre-existing primary vessels.

***Note:*** Newly formed vessels were defined as secondary or tertiary branches originating from pre-existing major vessels.

### Mouse subcutaneous Matrigel assay

**Timing: ∼10 days**

The following steps describe the detailed procedures for subcutaneous injection of the Matrigel solution into mice.

13. Animal preparation.
  a. Divide the mice into 5 groups, including three experimental groups, a negative control group, and a positive control group.
  b. Prepare the Matrigel mixture with final protein concentrations of 34.4 nM Pro-BMP9, 3.4 μM BMP9 precursor, 34.4 nM Pro-BMP9 plus 3.4 μM BMP9 precursor, and 44 nM FGF-2. Use FGF-2 as the positive control and PBS as the negative control (Figure 5A).

**Figure 5:**
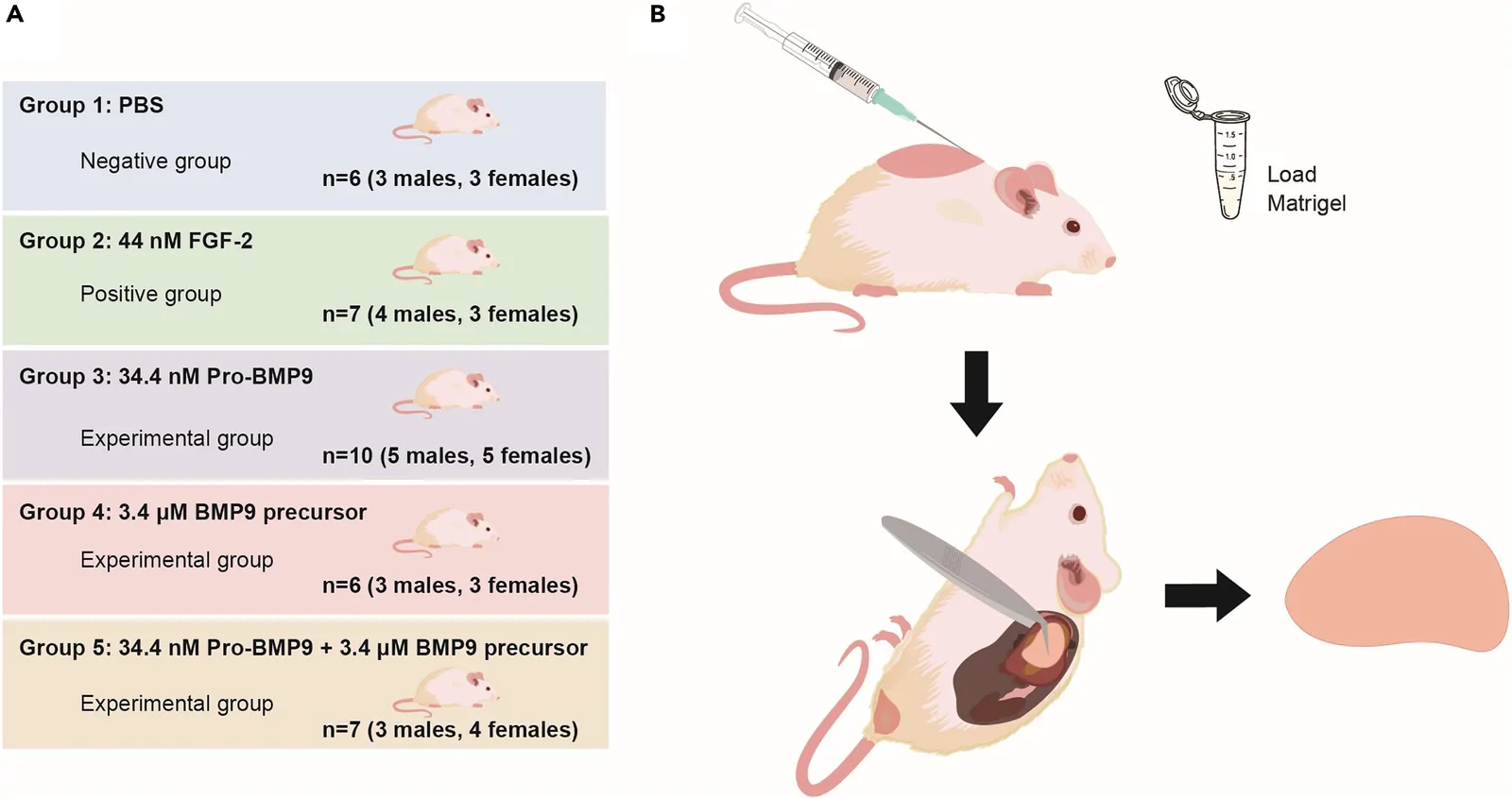
Schematic diagram of the experimental grouping of mice and workflow of the Matrigel plug assay (A) Mice were allocated to 5 groups as described in step 13. 450 μL Matrigel was mixed with 50 μL of PBS, 44 nM FGF-2, 34.4 nM Pro-BMP9, 3.4 μM BMP9 precursor or 34.4 nM Pro-BMP9 plus 3.4 μM BMP9 precursor at final concentrations, respectively, for the treatment of each mouse in each group. (B) The Matrigel mixture was injected into the subcutaneous tissue on the back of the mouse neck. After 1 week, the plugs were removed and processed for staining.
  c. Anaesthetize the mice with isoflurane anesthesia (induction at 3%–5% isoflurane and maintenance at 1%–2% isoflurane).
  d. Shave the injection site, and then swab the injection area with 75% ethanol for disinfection.

***Note:*** Assign the mice randomly to each group.

***Note:*** 40 BALB/c mice (19–21 g), including 20 females and 20 males, were received from the Institute of Radiation Medicine, Chinese Academy of Medical Sciences. In the preliminary experiment, 10 mice were divided into 5 groups: 2 (1 female and 1 male) in PBS group; 4 (2 females and 2 males) in Pro-BMP9 group; 2 (1 female and 1 male) in FGF-2 group; 1 male in the Precursor group; and 1 female in Pro-BMP9 plus the precursor group. In this experiment, some Matrigel plugs were crushed before immunostaining. After optimization, in the following experiment, 30 mice were divided into 5 groups with each containing 3 females and 3 males. All available results from these two experiments were reported. The final grouping information is: 6 (3 females and 3 males) in PBS group; 10 (5 females and 5 males) in Pro-BMP9 group; 7 (3 females and 4 males) in FGF-2 group; 6 (3 females and 3 males) in the Precursor group; and 7 (4 females and 3 males) in Pro-BMP9 plus the precursor group.

14. Inject the Matrigel mixture into the subcutaneous tissue of mice.
  a. Chill all equipment and reagents on ice before injection.
  b. Dilute the proteins to the desired final concentration in 50 μL PBS containing 40 U/mL sodium heparin and mix with 450 μL Matrigel solution on ice.
  c. Inject the Matrigel mixture (500 μL) into the subcutaneous tissue on the back of the mouse neck using a sterile 1 mL syringe fitted with a 27-gauge (0.45 × 16 mm) needle. See troubleshooting – problem 5.

***Note:*** Sway the needle back and forth after insertion to create a wide subcutaneous pocket before injecting the Matrigel mixture.

**CRITICAL:** It is necessary to pre-chill the Matrigel mixture as it will gel at the temperature above 10°C.

15. Remove the Matrigel plug from the mice.
  a. After 7 days post-injection, mice were euthanized by CO_2_ inhalation according to the regulations of the Research Ethics Committee.
  b. Remove the Matrigel plug with a surgical incision. Cut the tissue at the implantation site ∼5 mm wider on each side than the implantation area.
  c. Wash the Matrigel plugs with saline solution, quickly fix them with 10% formalin, and then store them at 22°C–25°C for one day (Figure 5B).
16. H&E staining.
  a. Embed the fixed Matrigel plugs in paraffin. Slice the embedded Matrigel plugs into 3 μm thick sections, attach them to glass slides, and bake the slides at 65°C for 2 h.
  b. Use xylene to wash the sections twice to deparaffinize, and rehydrate the sections with step gradients of ethanol (100–70%).
  c. Immerse the sections in Mayer’s hematoxylin solution for 10 min. Rinse them with water for 3 min.
  d. Immerse the sections in 1% acid alcohol (hydrochloric acid in 70% ethanol) for 30 s. Rinse them with water for 3 min.
  e. Stain the sections with eosin for 1 min and dehydrate them with step gradients of ethanol (80–100%).
  f. Immerse the sections in xylene for clearing and then mount them with neutral balsam under glass coverslips for microscopic examination.
17. Immunohistochemical staining.
  a. Slice the Matrigel plugs that were fixed with formalin and embedded in paraffin into 3 μm thick sections, attach them to glass slides, and bake the slides at 65°C for 2 h.
  b. Use xylene to wash the sections twice to deparaffinize and rehydrate them with step gradients of ethanol (100–70%).
  c. Perform antigen retrieval in 10 mM citrate and 1 mM EDTA (pH 9.0) using a pressure cooker. Cool for 1 h.
  d. Block the sections with 3% H_2_O_2_ for 15 min, wash them 3 times with PBS, and then block them with 3% goat serum.
  e. Label the sections with anti-CD31 antibody (1:2000) for 1 h, and then wash them 3 times with PBS.
  f. Label the sections with Envision-plus HRP-labelled Dextran polymer at 22°C–25°C for 30 min.
  g. Stain the sections with DAB staining solution and counterstain them with Mayer’s hematoxylin.
  h. Immerse the sections in xylene for clearing and then mount them with neutral balsam under glass coverslips for microscopic examination.

***Note:*** All antibodies used in immunohistochemical staining were diluted according to the manufacturer's protocol.

18. Quantify the H&E and immunohistochemically stained images using ImageJ.
  a. Click the icon to launch ImageJ.
  b. File > Open > Image > Type > 8-bit.
  c. Analyze > Calibrate > Uncalibrate OD.
  d. Image > Adjust > Threshold.
  e. Analyze > Set Measurements (Area, Integrated density, Limit to threshold).
  f. Analyze > Measure.
  g. Histological images were coded prior to analysis, and all measurements were performed by one investigator blinded to the treatment groups.

## Expected outcomes

The BMP9 precursor functions as an endogenous inhibitor to counteract the activity of Pro-BMP9. These assays demonstrate the angiogenic activity of Pro-BMP9 and confirm that the BMP9 precursor antagonizes the activity of mature BMP9 or Pro-BMP9.

Flow cytometric analysis is expected to show that the binding of Pro-BMP9 to its receptor is reduced upon addition of the BMP9 precursor, indicating that the BMP9 precursor and Pro-BMP9 compete with each other for receptor recognition. (Figure 6A) The expected outcome of the C2C12 differentiation assay is that Pro-BMP9 induces an increase in ALP activity in a dose-dependent manner, while the BMP9 precursor inhibits Pro-BMP9 activity in a dose-dependent manner, as presented by fitting the data to the Michaelis-Menten equation. (Figure 6B) In the CAM assay, Pro-BMP9 is expected to inhibit blood vessel sprouting. But the BMP9 precursor is expected to attenuate or even abolish this inhibitory effect. (Figure 6C) The mouse subcutaneous Matrigel assay will demonstrate that the BMP9 precursor antagonizes the activity of Pro-BMP9 in promoting vascular quiescence, which can be quantified by measuring CD31-positive area. (Figure 6D).

**Figure 6:**
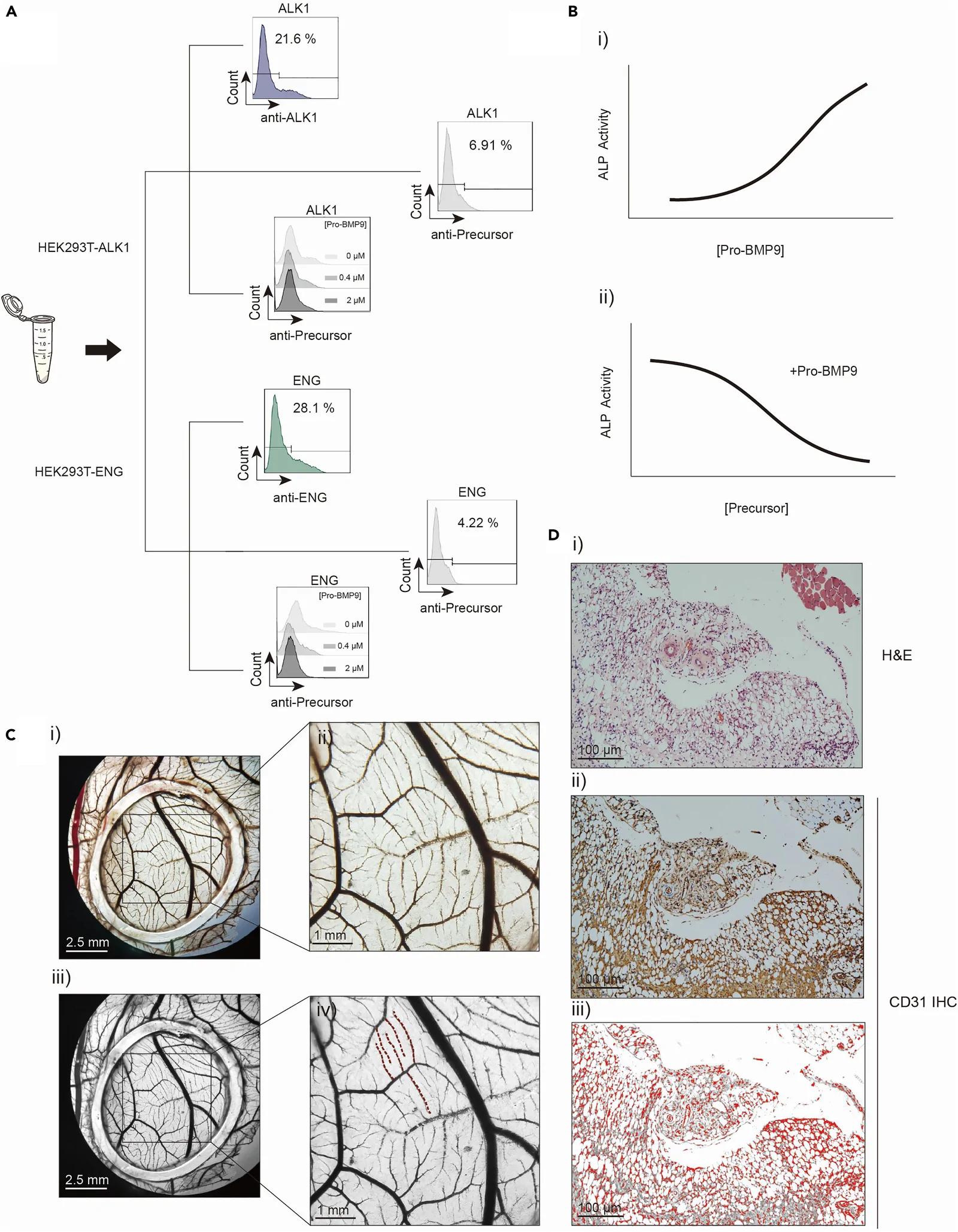
Expected outcomes showing that the BMP9 precursor acts as an intrinsic antagonist of its pro-protein in osteogenesis and angiogenesis (A) Pro-BMP9 competes with BMP9-precursor in binding to ALK1 and/or ENG expressed on HEK293T cells. (B) Schematic diagram of C2C12 differentiation assay, indicating that Pro-BMP9 induces osteogenic differentiation of C2C12 cells, while BMP9 precursor inhibits Pro-BMP9 signaling in C2C12 differentiation. (C) Overview of new vessel formation on the CAM treated with FGF-2 at day 11. Newly formed vessels are highlighted in red with scale bars of 2.5 and 1 mm. (D) Overview of H&E (i) and immunohistochemical staining (ii) of Matrigel plugs treated with FGF-2. The threshold regions (iii) are highlighted in red with scale bars of 100 μm. Figures were reprinted and adapted with permission from Zhang et al.^1^

## Limitations

This protocol provides detailed procedures for analyzing BMP9-induced angiogenic and osteogenic activities *in vitro* and *in vivo*. This protocol does not provide an evaluation of the dynamic association of the precursor with ALK1 or ENG. Furthermore, the CAM may exhibit hypersensitivity to the silicone ring used in the CAM assay, which needs to be considered during experimentation. Lastly, these *in vivo* or *in vitro* assays are not exhaustive for fully characterizing angiogenic and osteogenic potential of Pro-BMP9 and BMP9 precursor; thus, they should be combined with additional methods, such as SPR or BLI, to provide multi-dimensional validation.

## Troubleshooting

### Problem 1

Low survival rate of eggs.

### Potential solution

• Ensure continuous functioning of the egg incubator when incubating.
• Regularly monitor and calibrate the temperature and humidity of the incubator to maintain proper conditions.

### Problem 2

Low fluorescence intensity or low positive rate following the flow cytometric analysis.

### Potential solution

• Check the green fluorescence under a fluorescence microscope to verify the transfection efficiency, which usually needs to be higher than 60%.
• Use the mock-transfected cells as the negative control.
• Confirm that the 488 nm laser is on.

### Problem 3

Potential experimental variability caused by inaccurate approaches.

### Potential solution

• Load solutions slowly to avoid bubble formation.
• Calibrate pipettes to ensure technical consistency.

### Problem 4

Difficulty in forming an artificial air sac due to CAM perforation.

### Potential solution

• Pay attention to the piercing depth to avoid damaging the CAM.
• Apply sterile saline to the shell membrane to decrease the adhesion of the inner shell membrane, facilitating air sac formation.

### Problem 5

Failed to form a Matrigel plug in mice due to improper handling.

### Potential solution

• Submerge the Matrigel vial in ice and place it in a 4°C refrigerator for 14–16 h. Thaw before use. Avoid using Matrigel that contains particulate matter after thawing for 14–16 h.
• Ensure that the working concentration of the Matrigel solution is above 4 mg/mL to prevent incomplete gelation after injection.

## Resource availability

### Lead contact

Further information and requests for resources and reagents should be directed to and will be fulfilled by the lead contact, Li-Zhi Mi (lizhi.mi@tju.edu.cn).

### Technical contact

Technical questions regarding the experimental procedures described in this protocol should be directed to the technical contact, Li-Zhi Mi (lizhi.mi@tju.edu.cn).

### Materials availability

Plasmids generated in this study are available from the lead contact with a completed materials transfer agreement.

### Data and code availability

• All data reported in this paper will be shared by the lead contact upon request.
• This paper does not report original code.
• Any additional information required to reanalyze the data reported in this paper is available from the lead contact upon request.

## Acknowledgments

The authors are grateful to the financial support from the 10.13039/100014718National Natural Science Foundation of China to L.-Z.M. (#31670738 and #31470730) and X.Q. (#31400645).

## Author contributions

J.D., Y.Z., and W.Z. performed the experiments. X.Q. and L.-Z.M. supervised the experiments. J.D. drafted the manuscript. Y.Z., W.Z., X.Q., and L.-Z.M. revised the manuscript.

## Declaration of interests

The authors declare no competing interests.

## Declaration of generative AI and AI-assisted technologies in the manuscript preparation process

During the preparation of this work, the authors used ChatGPT to improve the language and readability. After using this service, the authors reviewed and edited the content as needed and take full responsibility for the content of the publication.
